# Effect of simvastatin on monocyte chemoattractant protein-1 expression in endometriosis patients: a randomized controlled trial

**DOI:** 10.1186/s12905-017-0446-3

**Published:** 2017-09-26

**Authors:** Wanwisa Waiyaput, Somphoch Pumipichet, Sawaek Weerakiet, Sasivimol Rattanasiri, Areepan Sophonsritsuk

**Affiliations:** 1 Office of Research Academic and Innovation, Faculty of Medicine, Ramathibodi Hospital, Mahidol University, Bangkok, Thailand; 2 Department of Obstetrics and Gynecology, Faculty of Medicine Srinakharinwirot University, Bangkok, Thailand; 3 Reproductive Endocrinology and Infertility Unit, Department of Obstetrics & Gynecology, Ramathibodi Hospital, Mahidol University, Rama VI Rd, Bangkok, 10400 Thailand; 4 Section for Clinical Epidemiology and Biostatistics, Faculty of Medicine, Ramathibodi Hospital, Mahidol University, Bangkok, Thailand

**Keywords:** Endometriosis, Simvastatin, Laparoscopy, Serum MCP-1, MCP-1 gene expression

## Abstract

**Background:**

Simvastatin is a promising new drug for the treatment of endometriosis. It is a cholesterol-lowering drug that acts by inhibiting HMG-CoA reductase, resulting in a decrease in mevalonate, a precursor of cholesterol and monocyte chemoattractant protein-1 (MCP-1). This study investigated the effect of pre-operative oral simvastatin administration on *MCP-1* gene expression and serum MCP-1 protein levels in patients with endometriosis.

**Methods:**

A prospective, randomized, controlled study was conducted at the Reproductive Endocrinology Unit of the Department of Obstetrics and Gynecology at the Faculty of Medicine Ramathibodi Hospital. Forty women (mean age: 18–45 years) scheduled for laparoscopic surgery who had been diagnosed with endometriosis were recruited and randomly assigned to either a treatment group (20 mg/d of orally administered simvastatin for 2 weeks before surgery) or an untreated control group. Serum was collected before and after treatment and protein levels of MCP-1 were determined. *MCP-1* and *CD68* transcript levels were also quantified using real-time PCR on endometriotic cyst tissues.

**Results:**

*MCP-1* gene expression on endometriotic cyst was not significantly different between the simvastatin-treated and untreated groups (*P* = 0.99). CD68 expression was higher in the treatment group compared to the control group, but this was not statistically significant (*P* = 0.055). Serum MCP-1 levels following simvastatin treatment were higher than in samples obtained before treatment (297.89 ± 70.77 and 255.51 ± 63.79 pg/ml, respectively) (*P* = 0.01).

**Conclusions:**

Treatment with 20 mg/d of simvastatin for 2 weeks did not reduce the expression of either the chemokine *MCP-1* gene or macrophage-specific genes. Cumulatively, this suggests that simvastatin is not ideal for treating endometriosis because a higher dose of simvastatin (40–100 mg/d) would be needed to achieve the target outcome, which would significantly increase the risk of myopathy in patients.

**Trial registration:**

Thai Clinical Trials Registry TCTR20130627003 Registered: June 27, 2013.

## Background

Endometriosis is a common gynecological disease among reproductive-age women. The prevalence of endometriosis is approximately 10% in reproductive-age women [1]. Endometriosis is defined as the presence of ectopic endometrial glands and stroma outside the uterine cavity [2, 3]. Clinical presentations include dysmenorrhea, chronic pelvic pain, dyspareunia, and infertility [3]. Diagnostic laparoscopy is the standard method used for the diagnosis of endometriosis [4]. Several theories have been proposed for the pathophysiology of the disease. These include retrograde menstruation, embryonic rests, and induction theory [5]. Although eutopic endometrium in most women sloughs through the patent fallopian tubes into the abdominal cavity during menstruation, it is unclear why endometrial tissues implant and grow in the peritoneal cavity only in some women. Recently, scientific evidence has suggested that immunological dysfunction may be associated with endometriosis [5, 6].

The immune cells present in peritoneal fluid are composed of macrophages, lymphocytes, mesothelial cells, and mast cells. The concentration of leukocytes is about 0.5–2.0 × 10^6^/ml, and approximately 85% of leukocytes are macrophages. The concentration of peritoneal macrophages in endometriosis patients appears to fluctuate during the menstrual cycle [6]. Macrophages likely play an important role in the production of peritoneal inflammatory factors, such as monocyte chemoattractant proteins (MCP), plasminogen activators, adhesion molecules, heme oxygenase, cytokines, and cyclooxygenase (COX)-2. Most of these inflammatory factors stimulate the process of endometrial adhesion and proliferation, as well as neovascularization [7].

Several medications, mainly hormones, are prescribed to treat endometriosis. However, patient tolerability rates are not high because of the side effects of these drugs. Novel alternative treatments for endometriosis have been proposed. For example, these include aromatase inhibitors, anti-angiogenic agents, anti-oxidants, and pro-apoptotic agents. Simvastatin, a cholesterol-lowering drug, has been recently proposed to treat endometriosis. It acts by inhibiting HMG-CoA reductase, resulting in a decrease in mevalonate, a precursor of cholesterol [8]. Aceto-acetyl-coA is metabolized by HMG-CoA reductase to mevalonate. The other beneficial effects of statin include anti-inflammation, decreased angiogenesis [9], decreased MCP-1, and matrix metalloproteinase-3 (MMP-3) [10]. However, the potential use of simvastatin for treating endometriosis has only been studied in vitro and in animals [9–14].

MCP-1 is a chemo-attractant for mononuclear phagocytes. It is one of the products of the mevalonate pathway and is secreted by white blood cells, macrophages, eutopic endometrial tissues, and endometriotic tissues [15, 16]. MCP-1 binds chemokine (C-C motif) receptors (CCR)-2 or CCR-4 to attract monocytes to target tissues. It promotes the migration of monocytes from the peripheral blood to the peritoneal cavity where they transform into macrophages and contribute to the local peritoneal inflammation commonly observed in endometriosis [15, 16]. For these reasons, MCP-1 may play an important role in the pathogenesis of endometriosis.

We hypothesized that orally administered simvastatin in patients with endometriosis might reduce serum MCP-1, endometriosis-associated MCP-1, and cluster of differentiation-68 (CD68), a surface marker for macrophages, expression. We conducted this prospective study to investigate the effect of oral simvastatin (20 mg/d) on CD68 and MCP-1 endometriotic cyst expression, and on serum MCP-1.

## Methods

This prospective, randomized, controlled study was conducted at the Division of Reproductive Endocrinology and Infertility in the Department of Obstetrics and Gynecology at the Faculty of Medicine Ramathibodi Hospital of Mahidol University in Bangkok, Thailand. It followed the recommendations of the Consolidated Standards of Reporting Trials (CONSORT-statement) for the design and reporting of randomized controlled trials. This study involving human subjects was approved by the Ethical Clearance Committee on human rights related to researches involving human subjects of Ramathibodi hospital. The study was registered with the Thai Clinical Trial Registry (www.clinicaltrials.in.th; registration number: TCTR 201306270 03).

### Study subjects

Subjects were women diagnosed with endometriosis requiring laparoscopic surgery. They underwent surgery between 30 June 2013 and 30 Jan 2014. Eligible women were aged 18–45 years and had experienced regular menstrual cycles for at least 3 cycles before surgery (cycle length ranged from 21 to 35 d during the 3 months before enrollment). Patients were otherwise in good health. The women were enrolled in the study if they had unilateral or bilateral ovarian endometriotic cysts confirmed by ultrasonography. All were willing to participate in the study, and signed an informed consent form. The subjects had not been previously treated with oral exogenous hormones or pills for at least 3 months. No patients had received statins, GnRH agonists, or DMPA within at least 1 year before surgery. None had contraindications to simvastatin. Exclusion criteria included pregnancy, side effects from oral simvastatin administration, allergy to the drug, and pathological outcomes not associated with endometriosis.

### Methods

Each surgery was scheduled to be performed in the early-to-middle follicular phase of the menstrual cycle. Participants were randomly divided into two groups during pre-operative evaluations. Serially numbered, opaque, sealed envelopes were obtained from a statistician using a computer-generated block randomization. These envelopes were opened by a physician at the outpatient department unit after patient enrollment.

**Fig. 1:**
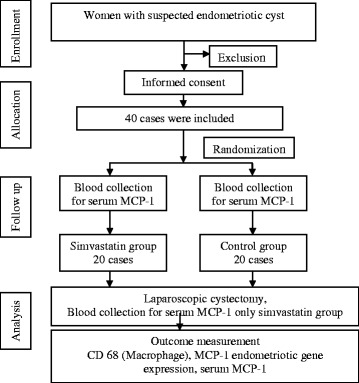
Study design for the effects of simvastatin on CD68 and MCP-1 expression level in endometrioma clinical trials

Forty eligible women signed informed consent forms to participate in the study. The participants were assigned to receive either oral simvastatin (20 mg/d) for 14 d before surgery (Group A, *N* = 20) or a placebo (control group, Group B, *N* = 20) (Fig. 1). After these assignments, all women had anthropometric measurements taken, including body weight and height measurements. BMI was calculated as the body weight in kilograms divided by a squared height in meters. Each participant was questioned about preoperative symptoms for a baseline assessment.

### Blood sample collection for MCP-1 immunoassay

Each patient was scheduled to give a blood sample during the early-to-middle follicular phase of the menstrual cycle. Blood samples were collected at the first appointment and before surgery for Group A. Blood samples were collected once before surgery for Group B. For each patient, a 10-ml blood sample was obtained from peripheral veins to measure the serum MCP-1 levels and liver enzymes. The blood was processed for analysis.

### Measurement of serum MCP-1

Serum MCP-1 levels were measured using a commercially available enzyme-linked immunosorbent assay kit (R&D Systems, Inc., U.S.A. Catalog # DCP00)**.**

### Endometriotic cyst tissue collection

Endometriotic cyst tissues were obtained during laparoscopic surgery. Briefly, the ovarian cyst was excised, and the dissection plane was meticulously identified. A thin layer of the cyst capsule was then extracted. Tissues of approximately 0.5 × 0.5 cm^2^ were placed in RNA*later*® solution for 24 h at 4 °C before storing at −80 °C.

### RNA isolation and quantitative real-time polymerase chain reaction

Total RNA was extracted from endometriotic tissues using the RNeasy Fibrous Tissue Mini Kit (Qiagen, Hilden, Germany). The RNA concentration and quality were determined by measuring the absorbance at 260 and 280 nm. Total RNA (1 μg) was reversed-transcribed to generate a cDNA library using an ImProm-II™ Reverse Transcription System (Promega). Quantitative real-time reverse transcriptase-polymerase chain reaction (RT-PCR) was performed on the CFX 96 Real Time PCR Instrument (Biorad, USA) using the SoFast ™ EvaGreen Supermix (Biorad, USA) and primers (Integrated DNA Technologies, Inc). The PCR reactions were performed for 35 cycles at 95 °C for 3 min, 59 °C for 5 s using primers specific for *MCP-1, CD 68, β-actin,* and glyceraldehyde-3-phosphate dehydrogenase (*GAPDH*). The primers used for amplification were: *MCP-1*: forward, 5΄- CCCCAGTCACCTGCTGTTAT-3΄, reverse, 5΄-TGGAATCCTGAACCCACTTC-3΄; *CD68*: forward, 5΄-ACTGAACCCCAACAAAACCA-3΄, reverse, 5΄-CGAGAATGTCCACTGTGCTG-3΄; *β-actin*: forward, 5΄-TCCTTCCTGGGCATGGAG-3΄, reverse, 5΄-GATGTCCACGTCACACTTCA-3΄; and *GAPDH*: forward, 5΄-TCTCTGCTCCTCCTGTTC-3΄, reverse, 5΄-ACCAAATCCGTTGACTCC-3΄. All RT-PCR data were normalized to the geometric mean of the endogenous control genes, *GAPDH* and *β-actin* to verify equal amounts of target cDNA in all samples*,* using the 2^−ΔΔCT^ procedure [17]. Serial cDNA dilutions were made to generate standard curves, and the gene-specific amplification efficiencies for each primer pair were determined.

### Statistical analysis

The planned sample size provided 80% power to detect an anticipated mean difference of at least 14.5% in the rate change of *MCP-1* gene expression for the pairwise comparisons between the arms of the two-sided 5% significance level by assuming an overall standard deviation (SD) of 15.3% and a loss of follow-up rate of up to 10%.

Statistical analyses were performed using STATA Statistical Software Version 12 (College Station, Texas: StataCorp. LP). Data were presented as the means ± SD and number (%). Median and percentile ranges (the 25th and 75th percentiles) were shown if the data were not normally distributed. The chi-squared or Fisher’s exact test was used to compare categorical variables. The student’s *t*-test, paired *t*-test, Wilcoxon-Mann-Whitney test, and sign-rank test were used to compare continuous variables. Kruskal-Wallis was applied to compare multiple non-normally distributed continuous variables. Pearson’s correlation coefficient was used to investigate whether there was a correlation between serum MCP-1 protein levels and *MCP-1* gene expression.

## Results

A total of 40 women were eligible for this prospective study. All participants were enrolled. Twenty subjects were assigned to the simvastatin group and the others to the control group. The clinical and baseline characteristics of the patients are summarized in Table 1. The mean age was 31 ± 5.21 and 30.7 ± 5.07 years in the control and simvastatin groups, respectively. No significant difference in ages between the groups was observed (*P* = 0.83). There were also no differences in BMI, parity, duration of menstruation, interval of menstruation, or infertility. The endometriosis stage according to the r-ASRM classification showed no differences between groups (*P* = 1.00). All cases were histologically confirmed for endometriosis.

**Table 1:** Baseline demographic data for women diagnosed with endometriosis treated with either simvastatin (simvastatin group) or placebo (control group)

Characteristics		Control group (*N* = 20)	Simvastatin group (*N* = 20)	*P* value
Age, mean ± SD (years)		31.05 ± 5.21	30.7± 5.07	0.83
BMI, mean ± SD (kg/m^2^)		20.91 ± 4.24	20.44 ± 3.43	0.71
Parity, N(%)	0	20 (100%) 0 (0%)	17 (88.24%) 3 (11.76%)	0.23
	1			
Duration of menstruation, mean ± SD (days)		4.4 ± 1.46	4.8 ± 1.11	0.36
Interval of menstruation, mean ± SD (days)		29.55 ± 1.73	28.8 ± 2.91	0.33
Infertility, N(%)	Yes	10 (50%) 10 (50%)	7 (47%) 13 (53%)	0.52
	No			
Stage of endometriosis, N(%)	III	7 (35%) 13 (65%)	7 (35.5%) 13 (65%)	1.00
	IV			

*BMI* body mass index

### *Endometriotic* MCP-1 *and* CD68 *mRNA expression after simvastatin treatment*

Relative *MCP-1* and *CD68* mRNA expression levels in endometriotic tissues were not significantly different between the simvastatin-treated and control groups (*P* = 0.99, *P* = 0.06, respectively) (Table 2). Moreover, the expression of these genes was not significantly different between the groups with or without deep infiltrating endometriosis (DIE) (Table 3).

**Table 2:** *MCP-1* and *CD68* gene expression levels in endometriotic cysts in the control versus simvastatin-treated group

Means ± SD	Control group (*N* = 20)	Simvastatin group (*N* = 20)	*P* value
Relative MCP-1 gene expression (×10^−2^)	3.68 ± 2.69	3.67 ± 3.15	0.99
Relative CD68 gene expression (×10^−2^)	6.87 ± 8.15	17.75 ± 23.19	0.06

*MCP-1* monocyte chemotactic protein-1

**Table 3:** *MCP-1* and *CD68* gene expression levels in endometriotic cysts in control versus simvastatin-treated patients with or without deep infiltrating endometriosis

	Relative MCP-1 expression (×10^−2^) Mean ± SD (*N* = 20)	*P* value	Relative CD68 expression (×10^−2^) Mean ± SD (*N* = 20)	*P* value
Control group		0.97		0.84
without DIE (*N* = 13)	3.85 ± 3.22		7.97 ± 9.22	
with DIE (*N* = 7)	3.38 ± 1.40		4.83 ± 2.24	
Simvastatin group				
without DIE (*N* = 14)	4.03 ± 3.63		15.01 ± 18.66	
with DIE (*N* = 6)	2.84 ± 1.46		24.15 ± 32.69	

*MCP-1* monocyte chemotactic protein-1, *DIE* deep infiltrating endometriosis

### Effect of simvastatin on peripheral MCP-1 levels before and after surgery

There was no statistically significant difference in serum MCP-1 levels during the follicular menstrual cycle at baseline between control and simvastatin-treated groups (*P* = 0.69). The mean levels of serum MCP-1 before and after simvastatin treatment were 255.51 ± 63.79 and 297.89 ± 70.77 pg/ml, respectively. There was also a significant difference in serum levels before and after treatment (*p* = 0.01). There was no correlation between the serum MCP-1 levels and *MCP-1* gene expression in patients after simvastatin treatment (*P* = 0.20*)* (data not shown).

## Discussion

In this study, we demonstrated that a single oral daily dose of 20 mg simvastatin did not reduce the expression of MCP-1 and CD68 in endometriotic cyst tissues after 2 weeks of drug administration. The presence of DIE had no effect on the outcome of simvastatin on the expression of target genes. No correlation between serum MCP-1 protein levels and *MCP-1* gene expression was found. However, serum MCP-1 levels increased after oral simvastatin administration. No adverse side effects were reported by patients after taking simvastatin.

The pathogenesis of endometriosis is multi-factorial. Retrograde menstruation is the most accepted theory for the development of endometriosis, and this idea was proposed by Sampson [18]. Local inflammatory responses and abnormalities in immunity also likely play important roles in the pathogenesis of endometriosis, specifically innate immunity [19]. The association between endometriosis, inflammation, and immunity has been well-recognized [20–24]. Moreover, immune dysfunction was theorized to be related to infertility problems experienced by patients with endometriosis, which has been demonstrated by the rejection of early implantation of embryos in women with infertility [25]. Macrophages, the major immune cells in the innate immune system, are the key cells contributing to the local inflammatory response in endometriosis. Monocytes are recruited to the endometriotic lesion by the chemotactic chemokine MCP-1, and they are then transformed into mature macrophages. Peritoneal macrophages secrete several cytokines regulating cell proliferation, activation, adhesion, chemotaxis, and transformation. Several cytokines are also released into peritoneal fluids, such as IL-1, IL-2, IL-6, IL-8, and TNF-α. These are all associated with the pathogenesis of endometriosis [19].

Medical treatments, such as hormonal treatments, have commonly been used to treat endometriosis. However, many patients cannot tolerate their adverse effects. Therefore, new approaches to endometriosis treatment have emerged, such as aromatase inhibitors, anti-angiogenic agents, anti-oxidants, and pro-apoptotic drugs [26]. Novel therapeutic strategies to reduce inflammation and/or immune responses have also been proposed. MCP-1 is a chemokine that creates the inflammatory environment associated with endometriosis, which induces monocytes to differentiate into macrophages and directs peripheral macrophages to the peritoneal cavity. Moreover, MCP-1 may stimulate endometrial cell proliferation, angiogenesis, and endometrial cell attachment. Therefore, targeting MCP-1 might be an effective way to inhibit inflammation, reduce cell-mediated immune responses, and cure endometriosis [19].

Statins are cholesterol-lowering drugs commonly used worldwide. They are potent inhibitors of 3-hydroxy-3-methyl-glutaryl coenzyme A (HMG-CoA), which inhibits MCP-1 production [8]. In vitro studies showed that simvastatin inhibits cell growth, cell proliferation, angiogenesis, endometrial cell attachment. It also induces apoptosis [9, 11–13]. In vivo studies by Bruner-Tran et al. evaluated the effects of simvastatin (5 and 25 mg/kg/d for 10 d) in a nude mouse model of endometriosis. They demonstrated a potent inhibitory effect of simvastatin on the development of endometriosis and on matrix metalloproteinase-3 (MMP-3) [10]. Cakmak et al. investigated the effects of simvastatin (5 and 25 mg/kg/d for 10 d) in a nude mouse model of endometriosis, and observed a significant decrease in MCP-1 production [27]. However, no studies have reported the effect of simvastatin on endometriosis in humans. The current research is a pioneering study that compares serum MCP-1 protein levels and *MCP-1* gene expression in women with endometriosis after oral administration of simvastatin. Our work is the second clinical trial of simvastatin for treating endometriosis. The first published clinical trial was conducted by Almassinokiani et al. (2013) [28]. Our findings showed that simvastatin administered at a dose of 20 mg/d for 2 weeks did not reduce *MCP-1* gene expression. The discrepancy among the in vitro studies, animal studies, and our human clinical trial could be explained by several reasons. The duration of treatment would not be one of these reasons because it seemed to be sufficient to reduce *MCP-1* gene expression. The duration of simvastatin treatment for the participants in the present study was longer (14 d) than that in the animal studies (10 d). The administration of simvastatin for 10 d in the nude mouse model for endometriosis created using human endometrium demonstrated the inhibitory effect of simvastatin on the size and number of endometriotic lesions [10] and on endometriotic MCP-1 protein expression levels [27]. However, the dose of simvastatin would be a significant factor influencing the medication’s effects. The result of treatment in the animal models in both studies showed a dose-response characteristic. A higher dose of simvastatin (25 mg/kg/d) achieved a better outcome than the lower dose (5 mg/kg/d) [10, 27]. The dose of simvastatin from nude mice to human or human equivalent dose (HED) was calculated using the following formula: HED (mg/kg) = animal dose (mg/kg) × animal Km / human Km. The Michaelis constant, Km, is the substrate concentration at which the reaction rate is half of Vmax. Vmax represents the maximum rate achieved by the system at maximum (saturating) substrate concentrations. The calculated dose of simvastatin in the present study was (5 × 3)/37 = 20 mg/d (if a woman’s weight averages 50 kg) [29]. We calculated the dosage of simvastatin based on an animal dose of 5 mg/kg/d. Therefore, according to the result from the animal studies, if we choose simvastatin with a higher dose, such as 25 mg/kg/d in mice model, we would probably observe a successful outcome. A simvastatin dose of 25 mg/kg/d in mice would be equivalent to 100 mg/d in humans. However, a dose of simvastatin higher than 80 mg/d is not recommended by the US FDA to prescribe to patients because of the high risk for myopathy [30]. In addition, these contradictory results could be caused by the different species and models used in the experiments.

Almassinokiani et al. (2013) conducted the first randomized clinical trial for simvastatin in women with endometriosis. They recruited 60 women diagnosed with endometriosis. These patients underwent conservative laparoscopic surgery and were then randomly assigned to receive either simvastatin (20 mg/d for 4 months) or GnRHa (Decapeptyl 3.75 mg IM each 28-d period for four doses). The authors demonstrated that treatment with simvastatin (20 mg/d for 4 months) reduced pain after conservative laparoscopic surgery to a similar extent as GnRHa treatment. However, many issues in the article were criticized. This included the blinding process for patients and physicians, and the allocation of the treatment modalities. In addition, women who wanted to conceive were more often chosen for simvastatin treatment than GnRHa treatment in order to avoid fetal abnormalities [31]. There were many differences between our study and Almassinokiani’s study. We compared simvastatin and no treatment for endometriosis before conservative surgery, while Almassinokiani et al. compared simvastatin and GnRHa treatments after conservative surgery. Moreover, the primary outcomes of our study and Almassinokiani’s study were different (MCP-1 expression vs. pain score). Almassinokiani’s outcome was more subjective than our primary outcome.

Simvastatin had no effect on macrophages in endometriotic tissue. We demonstrated that *CD68* gene expression in endometriosis was not affected by simvastatin administration. CD68 is a glycoprotein expressed in macrophage lineages. This result was supported by *MCP-1* gene expression analysis. No correlation between serum MCP-1 protein and *MCP-1* gene expression was demonstrated in our study. This suggests that MCP-1 is not only produced by endometrial cells, but also other cells, such as endothelial cells, fibroblasts, epithelial cells, smooth muscle cells, mesangial cells, astrocytes, monocytes, macrophages, mesothelial cells, and microglia.

One limitation of the present study is that we did not investigate peritoneal fluid MCP-1 levels. Although the primary source of MCP-1 is endometriotic cysts, it is possible that MCP-1 is produced by other cells. Assessing peritoneal fluid MCP-1 concentrations would also provide more information than serum MCP-1 levels alone. Future studies should focus on peritoneal fluid and cells. The stage of endometriosis development and the presence of DIE did not have any significant effect on the target genes of simvastatin. However, we did not analyze data from pelvic endometriotic tissues, endometriotic cysts, or DIE. Future studies will need to address this.

## Conclusions

We conclude that oral simvastatin (20 mg/d) does not reduce chemokine MCP-1 levels or macrophage gene expression in women diagnosed with endometriosis. A higher dose of simvastatin would promote favorable outcome for endometriosis patients. However, the higher the dose of simvastatin, the higher the risk of side effects, such as myopathy. Furthermore, serum MCP-1 is an unsuitable biomarker for predicting endometriosis.

## Abbreviations

CCR-2: Chemokine (C-C motif) receptors type 2
CCR-4: Chemokine (C-C motif) receptors type 4
*COX-2*: Cyclooxygenase type 2
*MCP-1*: monocyte chemoattractant proteins
*MMP-3*: matrix metalloproteinase-3
